# Blood parasite infection differentially relates to carotenoid-based plumage and bill color in the American goldfinch

**DOI:** 10.1002/ece3.1164

**Published:** 2014-07-23

**Authors:** David C Lumpkin, Troy G Murphy, Keith A Tarvin

**Affiliations:** 1 Department of Biology, Oberlin CollegeOberlin, Ohio; 2 Department of Biology, Trinity UniversitySan Antonio, Texas

**Keywords:** American goldfinch, bill color, blood parasites, carotenoid-based ornamentation, plumage color, *Spinus tristis*, *Trypanosoma*

## Abstract

Male and female American goldfinches (*Spinus tristis*) express condition-dependent carotenoid-based plumage and bill coloration. Plumage color is relatively static, as pigments incorporated into feathers during the spring molt cannot be mobilized thereafter. In contrast, bill color is dynamic, reflecting changes in condition over short time periods. Previous studies have shown that male and female ornaments, though similar in expression, are differentially related to measures of immunocompetence, suggesting that the relationship between ornamentation and parasite infection may differ between the sexes. In this study, we evaluate the relationship between condition-dependent ornamentation (plumage and bill color) and blood parasite infection in male and female American goldfinches. We captured goldfinches after completion of the pre-alternate molt and prior to the onset of nesting and assessed prevalence of *Trypanosoma* parasites via blood smears. Plumage color strongly predicted trypanosome infection: Birds with more colorful plumage were less likely to present infections. In contrast, we detected no relationship between infection and bill color, which in other studies has been shown to dynamically reflect current condition. Sex did not affect the relationship between infection status and either ornament. Together, these results suggest that physiological pathways linking carotenoid ornamentation and infection may vary even within a single species.

## Introduction

Ornamental signals used in social interactions often carry information about the condition of an individual (Zahavi 1975; Hamilton and Zuk 1982; Grafen 1990; Andersson 1994; Searcy and Nowicki 2005). Much research has focused on the hypothesis that carotenoid-based ornamentation is related to immune function and thereby serves as an honest signal of condition (e.g., Brawner et al. 2000; Dawson and Bortolotti 2006; Biard et al. 2009; Hill et al. 2009; Casagrande and Groothuis 2011). Results suggest that carotenoid-based signals are often honest indicators of health (Svensson and Wong 2011), either because there is a general trade-off between their use in immune function and signaling, or because carotenoid-based ornaments and immune function each are governed by a common set of biochemical and cellular processes that influence general condition (Hill 2011). Because of the physiological links between carotenoids and immunocompetence, carotenoid-based ornamentation can signal parasite infection (e.g., Brawner et al. 2000; McGraw and Hill 2000; Hill and Farmer 2005; Mougeot et al. 2009; del Cerro et al. 2010).

In many species, males and females exhibit similar ornamental expression (Tarvin and Murphy 2012), but similarly expressed ornaments may be under different selection pressures in the two sexes, possibly leading to different signaling function for similar ornaments (Amundsen 2000; Amundsen and Pärn 2006; LeBas 2006; Clutton-Brock 2007, 2009; Kraaijeveld et al. 2007; Murphy 2008; Tobias et al. 2012). In some cases, these differences may be attributed to genetic correlations between the sexes that give rise to mutual ornamentation without mutual function (Lande 1980; Muma and Weatherhead 1989; Cuervo et al. 1996; Wolf et al. 2004; Potti and Canal 2011). However, in other cases, it appears that ornaments have signaling function in both sexes (e.g., Amundsen et al. 1997; Velando et al. 2001; Jawor et al. 2004; Griggio et al. 2005; Siefferman and Hill 2005; Dakin 2011). Moreover, some studies indicate that the physiological basis of condition dependence may be sex dependent when ornaments are expressed similarly in both sexes, such that, for example, the same ornament relates differently to measures of immunocompetence in the two sexes (e.g., Potti and Merino 1996; Roulin et al. 2001; Lopez et al. 2008; Kelly et al. 2012; Zanollo et al. 2012). Differences between the sexes in how carotenoid-based ornaments relate to immunocompetence and infection could stem from differences in the ways that social and sexual selection processes affect males versus females, given the differences in the way fitness is maximized between the sexes (Lyon and Montgomerie 2012; Tobias et al. 2012).

**Figure 1:**
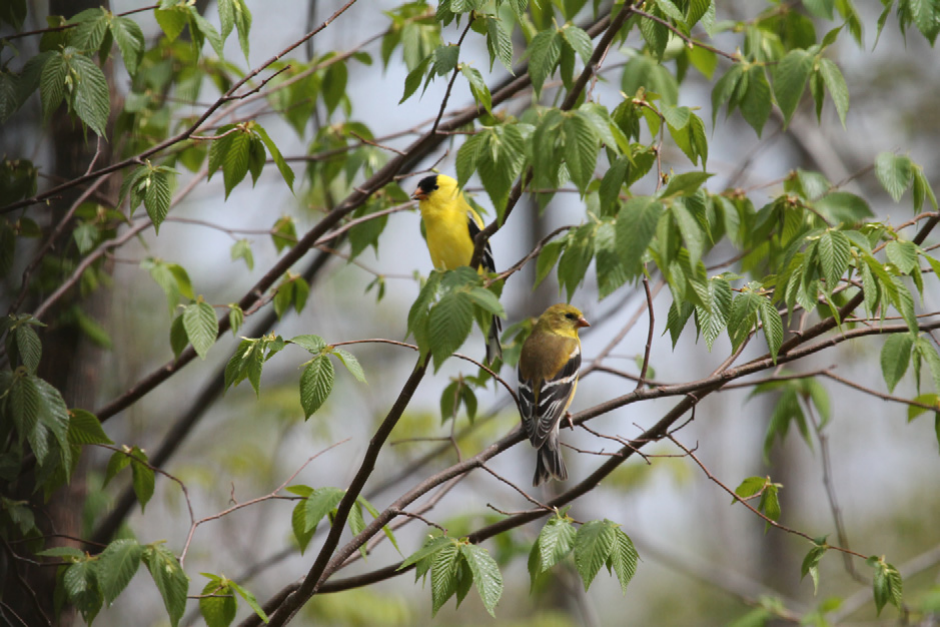
Male (left) and female American goldfinches. Both sexes express carotenoid-based yellow plumage and orange bills during the breeding season. Ornamental plumage is highly sexually dimorphic, whereas ornamental bill color is much less so. Photograph by Mary Garvin.

The American goldfinch (*Spinus tristis*; Fig. 1) serves as an excellent species with which to evaluate signaling in relation to parasite infection. They are sexually dimorphic songbirds in which carotenoid-based ornamentation is expressed in males and females. Following the pre-alternate molt that takes place during a roughly 6-week period in March–May, both male and female American goldfinches express patches of carotenoid-based yellow plumage and orange bills. Sexual dichromatism in plumage is pronounced, wherein the yellow plumage patches of males are far larger and tend to be more colorful (brighter, more saturated, and with a hue that is shifted toward longer, more orange, wavelengths; Kelly et al. 2012) than those of females. In contrast, the difference in bill color between the sexes is much less pronounced than plumage color, although bills of males tend to be brighter and slightly redder than female bills (Kelly et al. 2012). Goldfinches express sex-based differences in both immunocompetence and in the relationship between carotenoid-based coloration and immunocompetence: Kelly et al. (2012) found that females exhibited higher levels of both immunoglobulin Y (a component of acquired immunity) and natural antibodies (a component of innate immunity) than males and that those measures of immunocompetence were positively correlated with plumage and bill color, respectively, in females, but were unrelated to ornamentation in males. In previous work, McGraw and Hill (2000) showed that saturation of both plumage and bill color was reduced in captive male goldfinches that were infected with intestinal coccidian parasites during molt; however, it is unknown how parasitism affects female coloration. Thus, it is possible that parasitism may influence ornamentation differently in males and females of this species.

Trypanosomes (Kinetoplastida: Trypanosomatidae: *Trypanosoma*) are blood parasites found in a wide variety of bird species, and their prevalence can be substantial in bird populations (e.g., Bennett and Fallis 1960; Stabler et al. 1966). Although pathogenicity of trypanosomes appears to be relatively low in birds (Baker 1956), they have been shown to cause pathological effects in some species (e.g., Molyneux et al. 1983), and some studies have found a reduction in reproductive investment associated with trypanosome infection (Hakkarainen et al. 1998) or an increase in trypanosome prevalence or parasitemia in apparently immunocompromised individuals (Merino et al. 1996; Ilmonen et al. 1999; Valkiūnas et al. 2004). These patterns suggest that trypanosome infections may reflect immunocompetence, even if they do not impose direct pathological effects on their avian hosts (Apanius 1991). If so, then we may expect relationships between trypanosome infection and carotenoid-based ornamentation, since the latter frequently signals health (Svensson and Wong 2011).

In this study, we evaluate two components of signaling and trypanosome infection in American goldfinches. We first test whether plumage and bill color predict infection by trypanosome parasites, in accordance with the hypothesis that parasite infection either induces allocation of carotenoids from ornamentation to immune function, or that low-quality individuals or those in poor condition are less likely to both resist parasite infection and express elaborate ornamentation. Plumage and bill color differ in the temporal scale of their condition dependence: Plumage color reflects condition for approximately 5 months after molt (McGraw and Hill 2000, 2001), whereas bill color has the capacity to change within a few hours (Rosen and Tarvin 2006; Rosenthal et al. 2012) and can thus reflect condition dynamically. Second, because earlier work on this species has shown that male and female ornaments reflect different aspects of immunity (Kelly et al. 2012) and that the function of some ornamental traits differs between males and females (Murphy et al. 2014), we test whether the relationship between infection status and ornamentation differs between the sexes.

## Methods

In June and early July of 2010 and 2011, we captured adult goldfinches at Carlisle Reservation Metro Park in Lorain County, Ohio (41°17′22.33″N, 82°9′49.63″W). In 2011, we captured an additional four females and two males from a second metro park that was approximately 17 km distant from Carlisle Reservation (Wellington Reservation; 41°8′41.91″N, 82°14′6.30″W). We captured goldfinches in mist nets and basket traps at feeders containing nyjer (*Guizotia abyssinica*) seed. We measured bill and plumage color within 96 min of capture (mean ± SE = 23 ± 0.02 min) with an Ocean Optics USB4000 spectrometer with a PX2 xenon strobe lamp and a probe with a 3 mm aperture held 3 mm from the surface at a 90° angle, calibrating the spectrometer against a Spectralon white standard and a dark standard prior to measuring each body region. Four spectra were recorded from the bill (approximately 1 mm anterior to the nares) and the throat (approximately 5 mm anterior to the sternum) of each bird. We weighed birds to the nearest 0.1 g. We collected 20–80 *μ*L of blood from the brachial vein into heparinized capillary tubes. For each bird, we made two blood smears on microscope slides, which were subsequently air-dried. The remaining blood was used in another study. We determined sex and age from plumage (Pyle 1997). Birds were banded with uniquely numbered USFWS metal leg bands and released.

Following Rosenthal et al. (2012), we averaged the four spectral readings from each ornament of each individual bird and calculated tristimulus color variables using CLR version 1.05 and RCLR version 0.9.28 (Montgomerie 2008a,b). Briefly, brightness was calculated as the mean reflectance across the entire avian visual spectrum (320–700 nm, in 1 nm increments). Yellow saturation was calculated as the sum of reflectance between 550 and 635 nm, divided by the sum of reflectance across the entire spectrum. Hue was calculated as the wavelength in the 400–700 nm range corresponding to the midpoint of the highest and lowest reflectance values.

Blood smears were stained with 3 Step Stain Set (Richard-Allan Scientific, Kalamazoo, MI, USA) in 2010 and Harleco Hemacolor® (EMD Chemicals, Inc., Gibbstown, NJ, USA) in 2011. For each bird, we used the slide with the more uniform smear to assess trypanosome infection. The presence or absence of *Trypanosoma* was determined by scanning slides at 100 × for at least 5 min or until a *Trypanosoma* was found. We did not analyze intensity of *Trypanosoma* infection because there was little variation in the number of trypanosomes (1–3 per slide) among infected birds; moreover, because the remaining blood sample was used in another study, we were unable to use PCR to quantify infection intensity. Overall, infection status seemed to be reasonably consistent within individuals over the sampling period of our study, although some individuals appeared to gain or lose infection. Among 35 individuals that were captured twice within a season (typically sampled one to 2 weeks apart, all within the month of June), 71% had the same infection status at both captures. Among those birds that changed infection status, five changed from infected to apparently uninfected, and five changed from apparently uninfected to infected.

To avoid pseudoreplication, we used only the first capture of each individual in subsequent analyses; when an individual was captured in both years of the study, only a single randomly selected capture was included in analyses. Statistical analysis was conducted using IBM Statistics version 21 (SPSS, Armonk, NY, USA). We used principal components analysis (PCA) to collapse the three tristimulus variables into a single-color variable for throat plumage color and bill color, respectively, combining data from males and females such that the PCs could be directly compared between sexes. The resultant principal component for throat plumage color (hereafter, “throat color”) explained 76.2% of the variance and was positively correlated with throat hue (0.935), yellow saturation (0.959), and brightness (0.701); thus, higher values of throat color indicate more colorful (brighter, more saturated, greater hue) plumage. The principal component for bill color (hereafter, “bill color”) explained 45.2% of the variance and was negatively correlated with bill hue (−0.756) and positively correlated with saturation (0.807) and to a lesser extent, brightness (0.363). Because we wanted to control for sex-based variation in size (rather than test for its effects on infection), we regressed sex on mass to generate residuals as an index of relative mass for each (hereafter, “sex-adjusted mass”). Thus, positive values of sex-adjusted mass indicate a bird is heavier than the average of other birds of the same sex.

We used a backward elimination logistic regression procedure to test the hypothesis that ornamental coloration predicts parasite infection. The initial model included year, age, Julian capture date, sex-adjusted mass, sex, bill color, throat color, and the interactions between sex and bill color and sex and throat color. Probabilities of 0.05 and 0.10 were used as criteria for variable entry and removal, respectively.

The use of animals in this study was approved by the Oberlin College Institutional Animal Care and Use Committee (S10RBKT-13).

## Results

**Figure 2:**
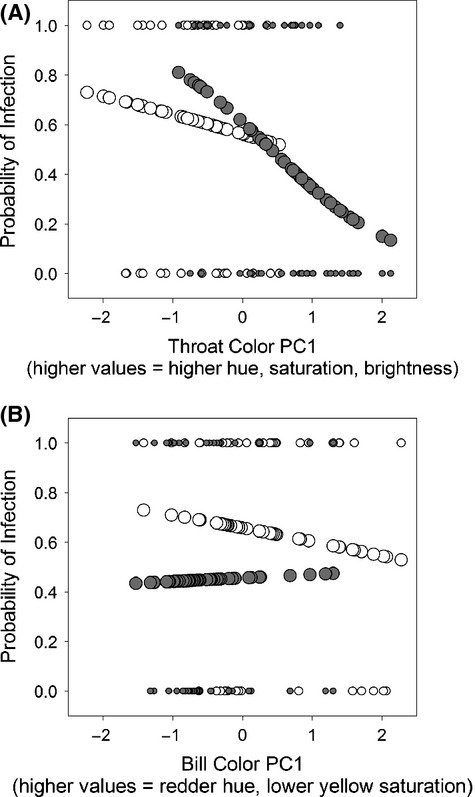
Relationship between *Trypanosoma* infection and (A) throat plumage color and (B) bill color in male and female American goldfinches. Open circles = females and filled circles = males. Small circles represent observed infection status (size of male circles is slightly smaller than female circles so that all data for all females can be seen in the graph). Large circles represent probability of infection predicted from logistic regressions in which either throat color or bill color was the sole independent variable; probabilities were generated for males and females separately. Note: predicted probabilities are rendered here only to illustrate the general relationship between ornaments and predicted probability of infection; the regressions from which these probabilities were derived did not take into account other variables that were included in the main analyses. Values for bill color PC1 were multiplied by −1.0 so that more colorful bills are on the right end of the *x*-axis.

We detected *Trypanosoma* in 21 of 48 male and 26 of 42 female goldfinches. Throat color significantly predicted trypanosome infection in our pooled dataset of males and females. Goldfinches with more colorful throat plumage (i.e., those with higher hue, yellow saturation, and brightness) were less likely to be infected with trypanosomes (effect on the model if throat color removed: change in −2 log likelihood = 8.424, df = 1, *P* = 0.004; Table 1, Fig. 2). Sex was not related to infection status, nor was the interaction between sex and plumage color (Table 2, Fig. 2). Neither bill color nor the interaction between bill color and sex was retained by the backward stepwise procedure, indicating they did not significantly predict infection status (Table 2, Fig. 2). Sex-adjusted mass also was significantly related to probability of infection, and Julian capture date was marginally related to probability of infection (Table 1).

**Table 1:** Backward elimination logistic regression analysis predicting trypanosome infection (presence or absence) in male and female American goldfinches. Variables retained in the final model are shown

							95% CI for Exp(B)	
							
Variable	B	SE	Wald	df	*P*	Exp(B)	Lower	Upper
Julian date	−0.069	0.040	3.019	1	0.082	0.934	0.864	1.009
Sex-adjusted mass	−0.737	0.347	4.513	1	0.034	0.479	0.243	0.945
Throat color	−0.674	0.245	7.551	1	0.006	0.510	0.315	0.824
Constant	11.371	6.472	3.087	1	0.079	86783.393		

**Table 2:** Variables eliminated from a logistic regression analysis predicting presence or absence of trypanosome infection in American goldfinches. The *t*-tests assessed whether the coefficient of the variable is different from zero. See text for details of the analysis

Variable	*t*	df	*P*
Bill color	0.988	1	0.320
Sex	0.286	1	0.593
Sex × Bill color	1.089	1	0.297
Age	0.564	1	0.453
Sex × Throat color	1.542	1	0.214
Year	2.601	1	0.107

To account for the fact that six individual birds were captured from the secondary study site, we re-ran the analysis without the birds captured at Wellington Reservation. The results were qualitatively identical except that year was marginally significant (*P* = 0.086), whereas Julian capture date was not retained in the final model.

## Discussion

Our results support the hypothesis that blood parasite infection is linked to lower carotenoid-based ornamentation in free-ranging American goldfinches. Specifically, we found a strong negative relationship between carotenoid-based plumage coloration and *Trypanosoma* infection status, suggesting that healthier birds had more colorful plumage. This pattern is consistent with results from other studies that investigated relationships between condition-dependent carotenoid-based ornamentation and parasite infection in birds (e.g., Brawner et al. 2000; McGraw and Hill 2000; Hõrak et al. 2001; Hill and Farmer 2005; Dawson and Bortolotti 2006; Baeta et al. 2008; Hill et al. 2009; Mougeot et al. 2009; Biard et al. 2010; del Cerro et al. 2010). In contrast, we did not find a relationship between bill color and trypanosome infection status. Our study adds to previous work on parasite-mediated signaling by investigating the influence of sex on the relationship between ornamentation and infection and by contrasting the influence of parasites on two very different carotenoid-based condition-dependent signal modalities – namely static plumage color and dynamic bill color, signals that differ substantially in the timescale over which they reflect condition and in their ability to track changes in condition in “real time.”

Our finding that plumage coloration predicted trypanosome infection suggests either that parasite infection interferes with carotenoid deposition in feathers during molt or that birds in poor condition are less able to both produce colorful plumage and resist trypanosome infection. Carotenoids may be used to relieve oxidative stress (McGraw 2011), suggesting there might be a physiological trade-off between the allocation of carotenoids toward plumage coloration and toward immune function (e.g., Blount et al. 2003; Alonso-Alvarez et al. 2004; Baeta et al. 2008; Pérez-Rodríguez et al. 2008). Indeed, circulating carotenoid levels have been found to be associated with immune function in other species (e.g., Faivre et al. 2003; McGraw and Ardia 2003; Aguilera and Amat 2007; Biard et al. 2009). Likewise, McGraw and Hill (2000) found that intestinal parasite infection during molt resulted in less saturated plumage color in male American goldfinches, suggesting that some parasites may directly interfere with carotenoid deposition in ornamental integuments. However, we measured parasite infection status several weeks after the spring molt had been completed, so it seems likely that the observed relationship between plumage color and infection may not involve a current trade-off (see Fitze et al. 2007; Hill 2011; see also Merino et al. 1996 for a discussion of elevation of trypanosome parasitemia in response to apparent immunosuppression). Our interpretation of these results is limited by the fact that we do not know when the birds were infected with trypanosomes relative to the time of sample collection and to time of molt (all our samples were collected in June or early July, whereas the bulk of molt occurs between late March and early May). Trypanosome infections in birds become patent in the blood within days of inoculation (Baker 1956; Bennett 1961), but they also can persist for months (Baker 1956; Woo and Bartlett 1982; Votýpka and Svobodová 2004) and new patent parasitemia can develop from latent infections many months later without new inoculation (Baker 1956; Valkiūnas et al. 2004). However, trypanosome prevalence tended to decline with date in our study, perhaps indicating that most of the infections we observed had become patent earlier in the season, closer to the molting period.

Previous work on a different population of American goldfinches found that female plumage color was positively correlated with natural antibody levels, whereas male plumage color was unrelated to measures of immunocompetence, suggesting that we might expect sex-specific differences in rates of parasitism or in the relationship between parasitism and ornament expression in this species (Kelly et al. 2012). However, results from the present study do not support either of these predictions: sex did not predict infection status, and the relationship between ornamentation and infection status did not differ between males and females. This suggests that even though males have much more extensive and more colorful plumage, the mechanisms linking plumage color and infection status appear to be similar in males and females.

In contrast to the signal properties of static plumage color, we detected no relationship in either sex between infection and bill color, an ornament that changes dynamically with short-term changes in physiological condition in both sexes (Rosen and Tarvin 2006; Rosenthal et al. 2012). If the presence of trypanosomes in blood smears is a strong index of current condition, we would expect a correlation between prevalence and bill color. One possible explanation is that the infections we observed may have been of low intensity and perhaps were not particularly costly during the sampling period. If so, the fact that plumage color was correlated with infection whereas bill color was not may indicate that plumage color better reflects the general (long-term) or past condition of goldfinches and that condition during March and April, when molt is occurring and hormonal shifts may compromise immunocompetence, influences both the development of ornamental plumage and the susceptibility or resistance to parasites. Bill color, on the other hand, may reflect other aspects of condition, such as short-term changes in stress (Rosenthal et al. 2012) which may not be directly linked to blood parasite prevalence measured after the period of initial infection. We expected bill color to signal parasite infection because Kelly et al. (2012) found that bill color of female goldfinches was predictive of more measures of immunocompetence (immunoglobulin Y and natural antibodies) than was plumage color, and because McGraw and Hill (2000) found that intestinal parasite infection during molt reduced bill saturation in male goldfinches. However, at present, we do not understand how measures of standing immunoglobulin Y and natural antibody levels relate to trypanosome infection. Clearly, more research needs to be conducted in order to understand the physiological links between condition and ornamentation in goldfinches.

This is the first study demonstrating a relationship between ornamentation and blood parasites in wild goldfinches, and likewise, it is the first to show a relationship between ornamentation and parasites in female goldfinches. Our findings indicate that trypanosome infection status is predicted by static condition-dependent plumage color, but not by dynamic condition-dependent bill color. This contrast in the relationship between infection and different carotenoid-based signaling modalities raises questions about the physiological mechanisms that link carotenoid ornamentation and infection, perhaps suggesting that multiple pathways may exist even within a single species.
